# Intersectionality in Weight Stigma Research: A Systematic Review of Empirical Evidence

**DOI:** 10.1007/s13679-026-00715-6

**Published:** 2026-04-15

**Authors:** Abdul-Hanan Saani Inusah, Menglin Shang, Xiaoming Li, Shan Qiao

**Affiliations:** 1 Department of Health Promotion, Education and Behavior, Arnold School of Public Health, University of South Carolina, Columbia, SC USA; 2 South Carolina SmartState Center for Healthcare Quality, Arnold School of Public Health, University of South Carolina, Columbia, SC USA

**Keywords:** Weight stigma, Intersectionality, Mental health, Discrimination, Health outcomes, Systematic review

## Abstract

**Background:**

Social stigma and discrimination based on body weight or size (weight stigma) contribute to inequities in healthcare access, yet research often treats weight stigma as a uniform exposure. Intersectionality has been invoked to address this limitation by situating weight stigma within intersecting systems of power and marginalization. However, how intersectionality is operationalized in empirical weight stigma research remains unclear.

**Objective:**

To examine how intersectionality is theoretically conceptualized and analytically operationalized in empirical studies of weight stigma, and to synthesize main intersectional findings.

**Methods:**

A PRISMA-guided systematic review was conducted using PubMed, Embase, PsycINFO, and Web of Science, with searches through December 12, 2024. Studies applying intersectionality in the context of weight stigma were included. Data were extracted on theoretical grounding, analytic strategies, measurement approaches, intersectional axes, outcomes, findings, and study quality.

**Results:**

Twenty-one studies met the inclusion criteria (10 qualitative, 11 quantitative). Analytic approaches to intersectionality varied across studies. Four themes emerged: co-occurring stigma across identities, context-specific manifestations, group differences in weight stigma, and cumulative intersectional disadvantage. Across these themes, weight stigma co-occurred with other forms of marginalization and varied across intersecting social identities, contributing to differences in healthcare access and everyday social experiences, with heterogeneous patterns observed across populations.

**Conclusions:**

Findings indicate that weight stigma operates through co-occurring systems of marginalization and varies across intersecting social identities, with important implications for health equity. However, approaches to operationalizing intersectionality remain inconsistent, highlighting a need for greater alignment between theoretical frameworks and analytic strategies.

**Supplementary Information:**

The online version contains supplementary material available at 10.1007/s13679-026-00715-6.

## Introduction

Weight stigma refers to social devaluation and denigration directed toward individuals because of their body weight or size, encompassing negative stereotypes, prejudice, and discriminatory treatment in interpersonal, institutional, and structural contexts [1, 2]. Weight stigma is increasingly recognized as a consequential driver of inequities in healthcare access and quality, contributing to differential treatment, reduced access to care, and poorer health outcomes for individuals with higher body weight [2, 3]. It has also been linked to healthcare avoidance and delayed preventive care, reinforcing inequities in healthcare utilization among individuals with higher body weight [4, 5]. Evidence suggests these patterns operate through anticipated judgment, prior stigmatizing clinical encounters, and healthcare environments that contribute to stress and disengagement among higher-weight patients [6–8]. Despite a growing consensus that weight stigma undermines health and healthcare equity, much of the empirical literature continues to conceptualize weight stigma as a largely uniform exposure, with limited attention to how its meanings, mechanisms, and consequences vary across social positions and lived contexts [1, 9].

Over the past decade, intersectionality has been increasingly invoked in health research as a necessary lens for understanding inequities that emerge from the interaction of multiple systems of power [10]. Methodological scholarship in public health has demonstrated that single-axis approaches can obscure meaningful heterogeneity in both exposure and outcomes, particularly in stigma research, where social hierarchies shape both interpersonal encounters and institutional practices [9]. At the same time, scholars have raised persistent concerns about how intersectionality is translated into empirical work, including inconsistencies in conceptual framing, analytic alignment, and attention to structural power [11, 12].

Despite increasing calls for intersectional approaches in obesity and health equity research, it remains unclear how intersectionality has been conceptualized and applied within empirical weight stigma research. Existing systematic reviews have primarily examined weight stigma within specific social groups, often organized around individual social categories or additive subgroup analyses rather than explicitly interrogating how intersecting systems of power jointly shape stigma processes [9, 12, 13]. As a result, it remains unclear the extent to which intersectional approaches have been intentionally operationalized in studies that explicitly state an intention to apply intersectionality.

This systematic review critically examines empirical studies that applied intersectionality in the study of weight stigma, with the following aims: (1) To examine how intersectionality is theoretically conceptualized within empirical weight stigma research; (2) To assess the analytic strategies through which intersectionality is operationalized across qualitative and quantitative study designs; (3) To characterize the contexts, outcomes, and key findings identified through intersectional analyses of weight stigma; and (4) To evaluate the methodological rigor and quality of studies applying intersectionality in weight stigma research.

This review synthesizes evidence across disciplines to demonstrate how intersectional approaches are applied in weight stigma research and how they shape understanding of variability in weight stigma experiences and their psychological, behavioral, and healthcare-related implications.

## Methods

The review was conducted and reported in accordance with the PRISMA guidelines [14], and the study protocol was registered with PROSPERO (registration number: CRD420251251092) in December 2025. All stages of literature search, data extraction, and quality assessment were conducted independently by two reviewers, with disagreements resolved through discussion and adjudication by a third reviewer when necessary.

### Search Strategy and Information Sources

A comprehensive electronic literature search was conducted in PubMed/MEDLINE, Embase, PsycINFO, and Web of Science. Searches were conducted from January 1, 2000 through December 12, 2025. The start date was selected because empirical applications of intersectionality within health research began to emerge primarily in the early 2000s.

The search string combined terms related to weight stigma (e.g., *weight stigma*, *weight bias*, *weight-based discrimination*), intersectionality (e.g., *intersectionality*, *intersecting identities*, *multiple stigma*), and health or healthcare–related contexts and outcomes. Searches incorporated both controlled vocabulary (e.g., MeSH and Emtree terms) and free-text terms. The full PubMed/MEDLINE search strategy is provided in Supplementary File 1. The search strategy was developed in consultation with an experienced health sciences librarian, reviewed by all authors.

### Eligibility Criteria

Studies were eligible for inclusion if they met the following criteria: (1) empirical studies (qualitative, quantitative, or mixed-methods) that examined weight stigma or related constructs, including weight bias, weight-based discrimination, or fatphobia; (2) explicitly stated an application of intersectionality, as articulated in the study aims, introduction, or analytic framing; and (3) peer-reviewed articles published in English. No restrictions were placed on population characteristics, geographic location, setting, or outcomes. Non-empirical publications, including reviews, commentaries, editorials, and protocols, were excluded.

### Study Screening

**Fig. 1:**
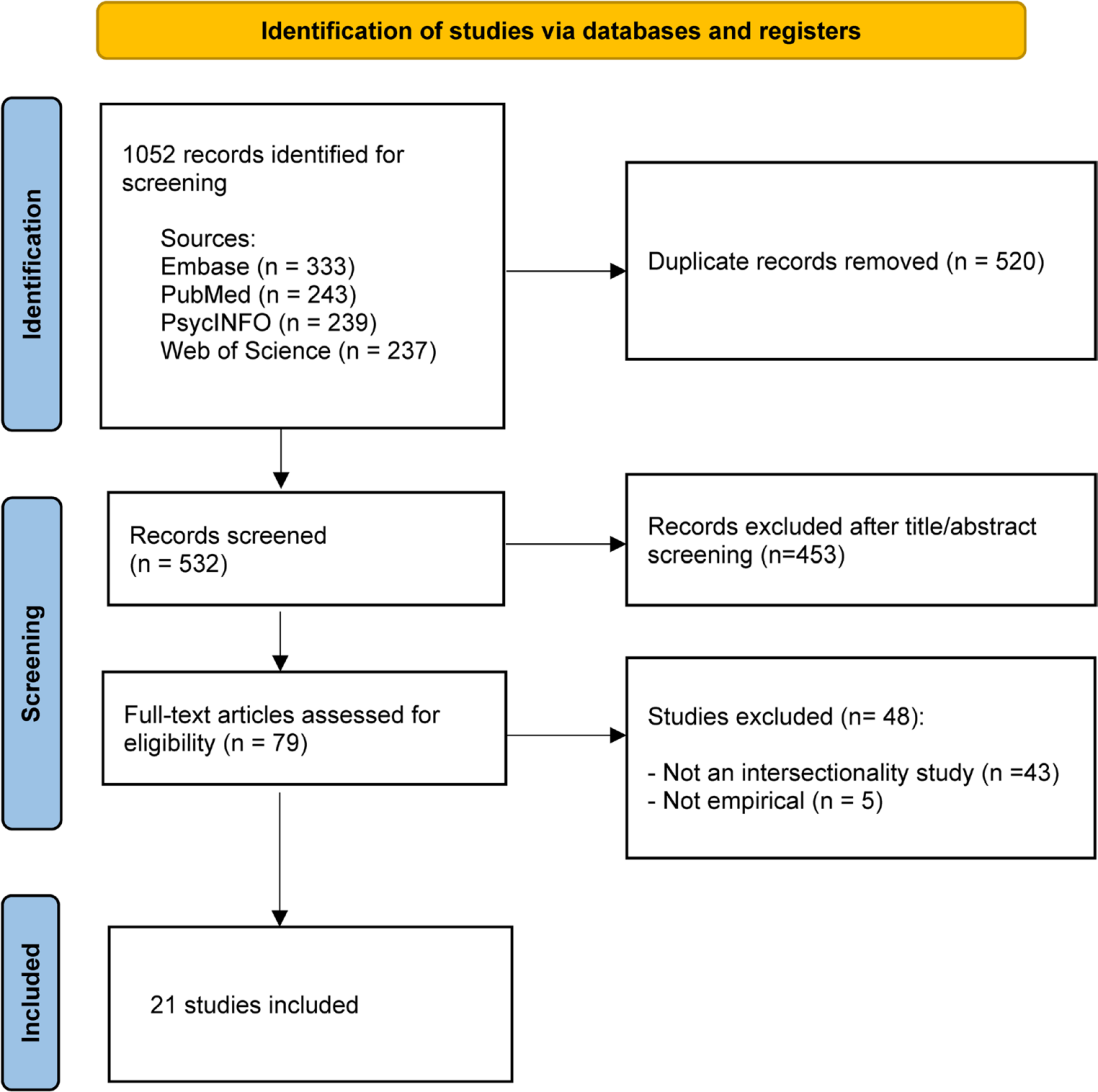
PRISMA flow diagram of study identification, screening, eligibility assessment, and inclusion

A total of 1,052 records were initially identified through database searches and imported into Covidence for screening management. Following removal of duplicates, 532 records remained for title and abstract screening. After initial screening, 79 articles were retrieved for full-text review and assessed in accordance with the eligibility criteria. After full-text assessment, 21 studies met the inclusion criteria and were included in the review. The study selection process is summarized in the PRISMA flow diagram (Fig. 1).

### Data Extraction

Data were extracted from all included studies using a structured extraction form developed by the review team. For each study, descriptive information was extracted on first author and year of publication, country, study population, recruitment setting or data source, study design, sample size, and key participant characteristics. Additional data included the theoretical framing used to situate intersectionality, the weight stigma constructs examined and measures employed, and the social axes included in intersectional analyses (e.g., race/ethnicity, sexual orientation). To capture how intersectionality was applied in empirical practice, extraction also documented analytic approaches used to operationalize intersectionality, including qualitative analytic methods and quantitative approaches such as interaction terms, latent class analysis, and other modeling strategies described by study authors. Finally, information was extracted on the outcomes or phenomena examined and the key intersectional findings reported.

### Study Quality Assessment

Methodological quality of included studies was assessed using appraisal tools appropriate to study design. Quantitative studies were assessed using the National Institutes of Health (NIH) Quality Assessment Tool for Observational Cohort and Cross-Sectional Studies [15]. Qualitative studies were assessed using the Critical Appraisal Skills Programme (CASP) qualitative checklist [16]. Quality appraisal results were used to contextualize findings during synthesis but were not used to exclude studies.

### Analytic Approach and Synthesis

An integrative narrative was used to synthesize findings across included studies. Extracted data were used to identify patterns and variation in how intersectionality was conceptualized, operationalized, and reflected in reported findings. Results are presented thematically, with supporting descriptive summaries provided in Tables 1 and 2.

**Table 1:** Descriptive characteristics of included studies

ID	Citation	Country	Study Population	Data Source/Recruitment Setting	Study Design	Sample size	Key Participant Characteristics
1	Adams, 2025	United States	Foreign-born Asian, Black, and Latinx young adults in the U.S.	Online national survey of racially diverse U.S. immigrants	Quantitative ross-sectional	200	Aged 18–39; 59% Asian, 15% Black, 27% Latinx; 40% men, 60% women; 26% self-identified overweight
2	Agénor et al., 2022	United States	Transmasculine young adults of color (ages 18–25)	Boston, MA; community organizations, health centers; in-person focus groups	Qualitative mini–focus groups	19 participants/5 focus interviews	Black, Latinx/e, Asian, multiracial; non-binary, transgender men; varied sexual orientations
3	Agénor et al., 2025	United States	Black and Latina cisgender and transgender women who use drugs	Boston (MA), Providence (RI), Washington DC; sexual and reproductive healthcare settings	Qualitative in-depth interviews	18	Mostly Black and Latina women; cisgender, transgender, and nonbinary identities; ages 23–45
4	Airhart-Larraga et al., 2025	United States	Latinx women who identify as larger-bodied	Online (virtual interviews), participants from TX, IL, CA	Qualitative phenomenological	6	All participants Latinx, female, larger-bodied; bachelor’s–doctoral education
5	Beccia et al., 2020	United States	Latinas aged 18–49	A Latina nationally representative survey (in-person interviews)	Quantitative cross-sectional	1,014	Majority first-generation immigrants; mostly Mexican, Puerto Rican, and other Latinx backgrounds; varied citizenship, Majority overweight/obese
6	Biefeld et al., 2025	United States	Gender-expansive adults (nonbinary, genderqueer, gender-fluid) and comparison group of cisgender adults	Online communities for LGTBQ and nonbinary individuals (Discord, Reddit, LGBTQ+ community orgs, college campus)	Qualitative	401	Majority white; includes nonbinary, gender-queer, gender-fluid, transgender; ages 18–65
7	Ciciurkaite & Perry, 2018	United States	Women aged 35–89	National Health Measurement Study (NHMS), national telephone survey	Quantitative cross-sectional	2,203	Mostly White, with Black and Hispanic women
8	Deol et al., 2024	Canada	Women of color with larger bodies within one year postpartum	Online (Zoom) interviews	Qualitative semi-structured interviews	10	Mostly Black/African American; one Hawaiian; mean ages 29.2 (± 3.0)
9	Elbe et al., 2024	United States	Larger-bodied White and Latino sexual minority men	Online Interviews	Qualitative interviews	9	*Mean* age = 37.89 (*SD* = 12.42), 5 White and 4 Latino/Hispanic, varied description of body size identities within sexual and social contexts
10	Fowler et al., 2025	United States	Cisgender non-heterosexual women	Online video-conference interviews (Zoom)	Qualitative life history-informed semi-structured interviews with reflexive thematic analysis	24	Cisgender women; diverse sexual orientations; majority non-Hispanic White, mean BMI 38.8 (SD = 13.6)
11	Garnett et al., 2014	United States	High school adolescents (9th–12th grade)	Boston public schools	Cross-sectional survey with latent class analysis	965	Majority Non-Hispanic Black and female; sexual minority youth included; overweight/obese and healthy-weight students represented (43.9%)
12	Gerend et al., 2022	United States	Cisgender women identifying as ethnic, racial, and/or sexual minority with high body weight and lived experience of weight discrimination	Zoom interviews	Qualitative semi-structured interviews	32	White, Black, and Latina women; over half sexual minority; BMI ranged from 31 to 64 kg/m2
13	Gerend & Ramirez, 2024	United States	Diverse U.S. adults aged 18–64 across the BMI spectrum	Online national panel survey	Quantitative cross-sectional	2,632	Half women; predominantly Black and Latino, 30% sexual minority, more than half overweight/obese
14	Harrop & Kattari, 2022	United States	Two, queer, nonbinary, chronically ill fat scholars	Academic/autoethnographic context	Qualitative autoethnography and auto-archaeology	2 authors (autoethnographic narratives)	Two fat, white, queer, nonbinary, chronically ill critical social work scholars
15	Himmelstein et al., 2017	United States	Adults aged 18+	National online survey panel	Quantitative cross-sectional	2,378	Half women, Majority White, mean BMI average BMI of 26.65 (SD = 5.74)
16	Launius & Launius, 2024	United States	Adults aged 21+	Online survey	Quantitative cross-sectional	397	Mostly White, women; includes Black, Asian, Hispanic/Latinx, and gender-diverse participants; mean BMI = 28.01 (SD = 7.61)
17	Makowski et al., 2019	Germany	Adults aged 18+	National telephone survey	Quantitative cross-sectional	692	Gender balanced; 55.4% overweight/obese, and 84.8% had contact to someone who is overweight
18	Osa et al., 2025	United States	Cisgender Latina and Hispanic women	Online survey	Quantitative cross-sectional	467	Varied ethnicities with nearly half being Mexican; 50.2% of participants had perceived their weight to be somewhat overweight or overweight
19	Paine, 2021	United States	LGBTQ patients	LGBTQ healthcare organization	Qualitative interviews	73 total (50 patients; 11 providers; 12 staff)	Diverse racial/ethnic identities; ciswomen, transmen, nonbinary
20	Panza et al., 2024	United States	Sexual minority women and heterosexual women with long-term weight loss	National Registry (online survey)	Quantitative cross-sectional	128 total (64 sexual minority; 64 heterosexual)	Mostly White women; sexual minority (lesbian, bisexual/pansexual, queer, asexual) matched to heterosexual women on BMI, age, race
21	Wang et al., 2024	United States	Youth in late childhood (9–12 years old)	National cohort from 21 sites, recruited through public and private schools	Quantitative longitudinal study (path analysis)	8,530	Diverse racial/ethnic (51.3% White), 9.2% gay or bisexual

**Table 2:** Empirical application of intersectionality in weight stigma research

Study	Theoretical Framework(s)	Sampling Strategy/Data Source	Weight Stigma Construct(s) and Measure(s)	Intersectional Axes Examined	Intersectionality Operationalization and Analytic Approach	Outcome(s) (Quantitative) or Phenomenon Examined (Qualitative)	Key Intersectional Findings
Adams 2025	Intersectional framework (Crenshaw, 1990; Himmelstein et al., 2017)	Online survey via Qualtrics panels	Internalized weight stigma (Modified Weight Bias Internalization Scale[WBIS-M])	Race, Marital status, Length of U.S. residence	Bivariate comparisons of weight stigma and psychological distress across individual identity categories (race, subjective weight status, length of U.S. residence, marital status) and multivariable regression models that incorporated demographic covariates	Internalized weight stigma & Psychological distress	High internalized weight stigma differed across race groups (Black: 34%; Asian: 9%; Latinx: 17%), across length of U.S. residence (≥ 10 years: 21%; <10 years: 10%), and across subjective weight status (perceiving oneself as overweight: 33%; not overweight: 9%). Severe psychological distress varied across subjective weight status (39% vs. 21%) and marital status (36% vs. 19%).
Agénor et al., 2022	Intersectionality framework (Black feminist theoretical framework)	Purposive sampling; recruited through community-based organizations, social media, and word of mouth	Qualitatively	Race/ethnicity; gender identity; disability/ableism; socioeconomic status	Qualitative thematic analysis guided by intersectionality; examined how multiple systems of oppression (cissexism, racism, ableism, weight-based discrimination) operated simultaneously in healthcare encounters	Experiences of discrimination in healthcare; impacts on healthcare access, quality of care, and patient well-being; strategies of resistance	Anti-Black racism intersected with cissexism and weight-based discrimination in healthcare for Black transmasculine participants. Black nonbinary participants described providers attributing their pain and symptoms to weight rather than investigating underlying causes. Anticipating stigma deterred some participants from seeking care. Participants also reported weight being used to delay or deny gender-affirming care.
Agénor et al., 2025	Intersectionality (rooted in Black feminist thought and praxis)	Stratified purposive sampling with quota	Qualitatively	Race/ethnicity; gender identity; sex assigned at birth; socioeconomic position; sexual orientation	Interview guide developed using an intersectional approach; focused on how intersecting systems of oppression are linked to experiences of sexual and reproductive health care	Experiences accessing and receiving sexual and reproductive health care	Fatphobia intersected with racism, sexism, and classism, contributing to assumptions about Black women’s intelligence and ability to understand SRH information, and shaping poor treatment in clinical interactions. Participants described being perceived as “an overweight Black girl from the South” and judged accordingly.
Airhart-Larraga et al., 2025	Intersectionality mentioned in background; no specific framework cited	Convenience sampling via social media (Facebook and LinkedIn) recruitment	Qualitatively	Gender; Latinx ethnicity	Intersectionality was referenced in the interview prompts to guide questions about how multiple identities shaped participants’ experiences	Lived experiences of sizeism; impacts on identity, emotional well-being, cultural expectations, and interactions within social and healthcare contexts	Participants described how being larger-bodied, female, and Latinx shaped experiences of sizeism through family weight-related messages, cultural food norms, and societal beauty standards. Some reported healthcare providers attributing health concerns to weight and making shaming comments. Acculturation influenced expectations about body shape and acceptance. Perceived judgments in public spaces reflected the interaction of size and gendered/Latinx beauty norms.
Beccia et al., 2020	Intracategorical intersectionality from Black Feminist scholarship	National Latino and Asian American Study (NLAAS), a four-stage probability sample of U.S. Latinx and Asian adults	Height/weight-based discrimination (measured via attribution on the Everyday Discrimination Scale)	Age; race; gender/sex; skin color; body size/weight (via discrimination attribution categories)	Intracategorical intersectionality used conceptually; analytic comparisons by discrimination attributions (e.g., height/weight, skin color, gender/sex, race, ethnicity, age). Modified Poisson models used to estimate associations	Lifetime history of recurrent binge eating	Height/weight-based discrimination was strongly associated with recurrent binge eating among Latinas, with an adjusted prevalence ratio of 10.24. Authors interpreted weight-based discrimination as an important contributor to binge-eating risk within Latinas’ intersecting social identities.
Biefeld et al., 2025	Tripartite Influence Model Featherstone’s consumer culture framework	Online recruitment via queer-friendly online communities (e.g., Discord, Reddit), community organizations, and a college campus	Qualitatively	Gender identity Race/ethnicity Body size/body shape Socioeconomic status/class	Qualitative thematic analysis grouping participants by gender identity and examining themes; intersectional considerations noted in thematic patterns	Body ideals, Body image experiences, Appearance expectations and pressures	BIPOC participants described thinness and whiteness as body ideals and reported pressures tied to race, queerness, and body size. Some Black participants discussed misgendering linked to feminine body shape and racialized expectations. Fatness was described as devalued and associated with whiteness-centered androgynous ideals.
Ciciurkaite & Perry, 2018	Modified labelling theory (Link et al., 1989).	National Health Measurement Study (NHMS), a national multi-stage probability sample via random-digit dial telephone surveys	Weight-based discrimination (survey attribution item; dichotomous indicator combining weight and appearance discrimination)	Race/ethnicity Socioeconomic status/class (education and income)	Multivariate regression including interaction terms between weight-based discrimination and race/ethnicity, income, and education; outcome examined separately across status groups	Psychological distress (aggregate mental health score)	Weight-based discrimination had a stronger negative association with mental health among Hispanic women; no significant difference was found between Black and White women. Lower-income women experienced larger declines in mental health in response to weight-based discrimination, while higher-income women showed minimal change.
Deol et al., 2024	Intersectionality (Crenshaw), Health Stigma and Discrimination Framework	Recruitment via social media (X(Twitter) and Facebook); participants completed an online eligibility survey in Qualtrics and were scheduled for semi-structured interviews.	Qualitatively	Race/ethnicity Body size (larger bodies)	Interview guide included a question asking participants to reflect on how racial/ethnic background influenced their views on body weight; qualitative thematic analysis included an intersectional experiences theme	Experiences of weight stigma during pregnancy	Participants described racialized differences in how weight gain is perceived, noting that Black women may be expected to carry more weight than White women. Some participants referenced cultural or geographic background as shaping expectations of body size and weight changes in pregnancy.
Elbe et al., 2024	Intraminority stress theory; sexual field theory.	Social media recruitment (Twitter, Instagram, Reddit and Facebook)	Qualitatively	Sexual orientation	Intersectionality was used to interpret how weight stigma was experienced, with attention to how body size intersected with sexual identity in shaping stigma, desirability, and coping strategies.	Lived experiences of weight stigma and stigma resistance in casual sexual encounters	Participants described weight stigma within dating and sexual contexts, where fatness shaped experiences of desirability, rejection, and fetishization. Masculinity norms within gay male communities influenced how body size was evaluated, and participants described navigating stigma through partner vetting and selective engagement.
Fowler et al., 2025	Intersectionality theory (Crenshaw, 1989), Minority stress theory, Life course perspective	Purposive sampling using maximum variation strategy to recruit diverse participants via social media posts (Facebook and Instagram) linking to an online screening survey	Qualitatively	Sexual orientation Race/ethnicity Body size Disability status Socioeconomic position/class Gender presentation	Interviews examined experiences of weight stigma over time; results described intersectional experiences across identities using reflexive thematic analysis.	Experiences of weight stigma across the life course	Participants described weight stigma occurring alongside sexual orientation, race/ethnicity, and disability status. Some participants described being fat and queer as shaping body expectations and acceptance within queer communities, and being fat and disabled as shaping stereotypes and assumptions encountered in healthcare settings.
Garnett et al., 2014	Intersectionality (Crenshaw, 1989)	Two-stage stratified random sampling via the Boston Youth Survey	Weight-based discrimination (single attribution item)	Race/ethnicity; immigration status; weight	Latent class analysis used to model co-occurring attributes of discrimination and bullying; intersectionality explicitly informed class construction and interpretation.	Depressive symptoms; deliberate self-harm; suicidal ideation	A latent class characterized by co-occurring racial, immigration, and weight-based discrimination with bullying/assault had higher odds of deliberate self-harm and suicidal ideation compared to the low discrimination class.
Gerend et al., 2022	Intersectionality (Crenshaw, 1991; Bowleg, 2012). Theories of discrimination and health (Gallo et al., 2009).	Purposive sampling (maximum variation sampling) via Facebook advertisements	Qualitatively	Race; ethnicity; sexual orientation; gender	Intersectional lens guided study design, interview guide, and thematic analysis; intersectional experiences were described across themes in results.	Experiences of weight discrimination and factors shaping vulnerability or resilience to its harmful health consequences	Participants described that experiences of weight discrimination differed depending on intersections of weight with race, ethnicity, and sexual orientation, particularly through norms around femininity and attractiveness.
Gerend et al., 2024	Theories of internalized weight stigma and health (Ratcliffe & Ellison, 2015; Sikorski et al., 2015); intersectionality theory (Crenshaw, 1991;Bowleg, 2012).	Quota-based sampling via a national U.S. online panel (Dynata)	Internalized weight stigma (modified Weight Bias Internalization Scale [WBIS-M])	Gender; race; ethnicity; sexual orientation	Two- and three-way interaction terms among gender, race, ethnicity, and sexual orientation were tested in ANCOVA models.	Internalized weight stigma (WBIS-M score)	Internalized weight stigma(IWS) differed by race × gender and race × ethnicity interactions. Non-Black women reported higher IWS scores than Black women, while Black and non-Black men had similar scores. Among non-Latino respondents, Black respondents reported lower IWS scores than non-Black respondents; scores were similar for Black and non-Black respondents who identified as Latino.
Harrop & Kattari, 2022	Social Identity Threat Theory (Major & O’Brien, 2005; Hunger et al., 2015)	Autoethnographic and auto archaeological self-study by the authors	Qualitatively	Body size/gender identity; sexual orientation; race; disability status; chronic illness; class	Intersectional identities explicitly centered through identity-centered autoethnographic and auto archaeological analysis; intersections described across narratives and interpreted through Social Identity Threat Theory	Experiences of coming out as fat and navigating weight stigma across intersecting identities	The authors narrated how experiences of weight stigma were shaped by intersections of fatness with gender identity, sexual orientation, disability, chronic illness, and class, particularly in healthcare, educational, and professional settings. They described how these intersecting identities influenced recognition of stigma, access to care and accommodations, decisions about disclosure, and strategies for navigating weight stigma.
Himmelstein et al. 2017	Intersectionality (Crenshaw, 1991)	National survey panel sample recruited via Survey Sampling International with quota-based sampling.	Experienced weight stigma (binary measure); weight bias internalization (Modified Weight Bias Internalization Scale [WBIS-M])	Race; gender	Intersectionality examined through race × gender interaction terms in regression models assessing experienced stigma, weight bias internalization, and coping strategies	Experienced weight stigma; weight bias internalization; coping strategies	No race or gender differences were observed for experienced weight stigma. Weight bias internalization differed by race × gender, with Black men and women reporting lower internalization than White men and women, and the effect stronger among Black women. Coping strategies varied by race × gender: Black women were less likely than White women to cope via disordered eating, Hispanic women were more likely than White women to cope via disordered eating, and Black men were more likely than White men to cope via eating.
Launius & Lydecker, 2024	Not reported	Online recruitment platform (Mechanical Turk) through an advertisement	Weight-based discrimination (Everyday Discrimination Scale; Weight self-stigma (Weight Self-Stigma Questionnaire); Internalized weight bias (modified Weight Bias Internalization Scale[WBIS-M])	Gender; race/ethnicity; age; physical appearance	Interaction terms tested between weight-based discrimination and other attributed reasons (gender, race/ethnicity, age, physical appearance) in ANOVA models	Eating-disorder psychopathology; weight self-stigma; internalized weight bias; perceived stress; depressive symptoms; self-rated general health	Weight-based discrimination combined with gender, race/ethnicity, age, or physical appearance was not associated with worse outcomes than weight-based discrimination alone.
Makowski et al., 2019	Not reported	Representative cross-sectional telephone survey using random digit dialling (landline and mobile)	Fat phobia (Fat Phobia Scale)	Gender; socioeconomic status (SES)	Main effects of gender and SES and their interaction (gender × SES) tested in multiple linear regression models using vignette-based stigma outcomes	Fat phobia; negative emotional reactions; positive emotional reactions; desire for social distance	Male gender and low SES in the obesity vignette were each associated with higher fat phobia and greater desire for social distance, and male gender was associated with more negative emotional reactions; no significant gender × SES interaction effects were observed.
Osa et al., 2025	Intersectionality (Crenshaw, 1991)	Online panel recruitment via Qualtrics Panels	Perceived weight status (single-item self-classification)	Racial Discrimination Stress (RDS)	Hierarchical linear regression models testing main effects of racial discrimination stress and perceived weight status, and their interaction (racial discrimination stress × perceived weight status) on depressive symptoms	Depressive symptoms	Racial discrimination stress and perceived weight status were each independently associated with depressive symptoms. The interaction between racial discrimination stress and perceived weight status was not significantly associated with depressive symptoms.
Paine, 2021	Not reported	Patients recruited from an LGBTQ-focused primary care clinic	Qualitatively	gender identity; sexual orientation; race	Qualitative analysis of patient interviews examining weight stigma across multiple identities.	Experiences of weight bias and how weight stigma shapes healthcare interactions.	Weight stigma in healthcare was experienced as intersectional stigma co-occurring with gender identity, sexual orientation, race, disability, and chronic illness, and was associated with barriers to care including acute distress, reduced patient–provider communication, avoidance of disclosure, reduced adherence, and delayed or foregone care-seeking, contributing to sexual and gender minority health disadvantage.
Panza et al., 2024	Minority Stress Theory; Intersectionality Theory (Crenshaw, 1991)	Registry-based survey sample from the National Weight Control Registry	Experienced weight stigma (past-year weight-based negative treatment); Internalized weight stigma (Modified Weight Bias Internalization Scale[WBIS-M]); Weight self-stigma (Weight Self-Stigma Questionnaire [WSSQ])	Sexual orientation	Group comparisons of sexual minority women versus heterosexual women on weight stigma outcomes using generalized linear models; models adjusted for age and BMI	Experienced weight stigma; internalized weight stigma; weight self-stigma	Sexual minority women had a higher prevalence of past-year experienced weight stigma and higher levels of internalized weight stigma, including clinically significant internalization, compared with heterosexual women matched on age and BMI.
Wang et al., 2024	Theory of Planned Behavior	National, multi-stage probability school-based sample from the Adolescent Brain Cognitive Development (ABCD) Study	Weight-based discrimination (binary items); multiple discrimination (count of discrimination due to race/ethnicity, sexual orientation, and weight)	Race/ethnicity; sexual orientation	Youth were categorized into non-marginalized, single marginalized, and intersecting marginalized identity groups based on race/ethnicity, sexual orientation, and overweight status; cross-lagged path models tested additive, multiplicative, and inuring effects of multiple discrimination on substance use intention, with subgroup sensitivity analyses.	Substance use intention	Youth with intersecting marginalized identities reported higher levels of multiple discrimination and substance use intention than non-marginalized and single-identity youth; in longitudinal analyses, multiple discrimination predicted higher subsequent substance use intention only in this group, while substance use intention predicted lower subsequent discrimination.

## Results

### Overview of Included Studies

This review included 21 studies, of which 19 were conducted in the United States, 1 in Canada, and 1 in Germany. Study designs included 10 qualitative studies and 11 quantitative studies, of which 10 were cross-sectional studies and 1 was longitudinal. Qualitative studies primarily relied on in-depth interviews or focus group discussions.

Quantitative studies included sample sizes ranging from 128 [17] to 8,530 [18], while qualitative studies typically included 6 [19] to 73 participants [20], with the exception of one autoethnographic study involving two authors’ self-reflections [21].

Sampling strategies and data sources varied across studies. Quantitative studies most commonly used national probability surveys [22–24], online panel surveys [25, 26], registry-based samples [17], or school-based cohorts [18, 27] while qualitative studies primarily relied on purposive, maximum variation, or convenience sampling, as reported by study authors. Several qualitative studies recruited participants through healthcare clinics [28], community-based organizations [29], or LGBTQ-focused organizations [20], while others used online recruitment via social media platforms such as Facebook and X [30, 31].

Study populations included children and adolescents, adults across the BMI spectrum, postpartum women, and groups defined by race and ethnicity, gender identity, sexual orientation, or body size. One qualitative study recruited multiple participant groups such as patients, healthcare providers and staff [20]. Weight stigma was examined across a range of contexts, including healthcare settings, schools, public and social spaces, and general societal contexts (see Table 1).

### Quality Appraisal of Included Studies

Among quantitative studies, four were rated as good, six as fair, and one as poor. Overall quality ratings are summarized in Supplementary Table S1. Quality appraisal of qualitative studies indicated that all clearly articulated research aims, employed appropriate qualitative methodologies, and reported findings that addressed their stated research objectives. Most demonstrated adequate rigor in data analysis and reflexivity and addressed ethical considerations. CASP appraisal results are summarized in Supplementary Table S2.

### Weight Stigma Constructs or Measurement

All qualitative studies examined weight stigma through participants’ narratives, rather than standardized scales. Across these studies, authors used terms such as weight stigma, sizeism, fatphobia, or anti-fatness to describe lived experiences and social interactions identified through interview data and thematic analysis.

Across qualitative studies, weight stigma was consistently characterized as a relational and context-dependent social process rather than a singular exposure [20, 21, 28]. Participants described it as enacted through interpersonal interactions, institutional encounters, and cultural norms, often alongside other forms of marginalization such as racism, cissexism, and ableism [28, 32]. Commonly reported manifestations included attribution of health concerns to body weight, moral judgments about personal responsibility, and exclusion within healthcare, family, social, and community settings. These constructs were articulated using varied terminology but were consistently grounded in lived experiences of social devaluation and unequal treatment [19, 30].

Among the quantitative studies, weight stigma was most commonly operationalized as experience of weight-based discrimination. Nine studies examined experienced discrimination, typically measured using single-item attribution questions [23], binary indicators of unfair treatment [1], or adaptations of the Everyday Discrimination Scale that included weight as an attributed reason [24].

Internalized weight stigma was examined in five quantitative studies. Four studies measured internalized weight stigma using the modified Weight Bias Internalization Scale (WBIS-M) [1, 25], while one study used the Weight Self-Stigma Questionnaire (WSSQ) [33]. In some studies, internalized weight stigma was examined alongside measures of experienced weight-based discrimination [1, 17, 25]. A small number of quantitative studies examined weight stigma as part of broader measures of multiple forms of discrimination, in which weight-based discrimination was assessed alongside other forms of discrimination, including race or ethnicity and sexual orientation [18].

One quantitative study examined public obesity stigma using vignette-based measures, in which respondents were randomly assigned to audio vignettes depicting individuals with obesity that varied by gender and occupational position as a proxy for socioeconomic status, and stigma was assessed using standardized scales capturing fat phobia, emotional reactions, and desire for social distance [22].

### Conceptualization of Intersectionality in Weight Stigma Studies

Many studies explicitly cited intersectionality theory, most commonly drawing on Crenshaw’s formulation of intersectionality [1, 26, 27, 32, 34, 35]. A smaller number of studies explicitly referenced Black feminist scholarship as the primary theoretical grounding for their intersectional approach [24, 28, 36]. Several studies cited intersectionality alongside additional theoretical perspectives, including minority stress theory [17], life course perspectives [32], social identity threat theory [21], and theories of discrimination and health [34].

Across studies that cited intersectionality, authors generally treated it as a framework for examining intersecting social identities and systems of oppression, including race or ethnicity, gender or gender identity, sexual orientation, socioeconomic position, disability or chronic illness, and body size or weight. A small number of studies referenced intersectionality without citing a specific theoretical framework, using it to signal attention to multiple social identities rather than articulating a theoretically grounded intersectional approach [19, 20, 22, 33].

### Intersectional Axes Examined Across Studies

Race and/or ethnicity were the most frequently examined intersectional axes and were included in 18 of the 21 studies (see Table 2). These studies examined weight stigma among racially and ethnically diverse populations or tested differences across racial and ethnic groups. Gender-related axes, including gender, gender identity, sex assigned at birth, or gender presentation, were examined in 12 studies. Several studies focused on cisgender women, transgender men, or gender-diverse populations, while others examined gender as a binary variable in quantitative analyses.

Sexual orientation was examined in 9 studies, including studies of sexual minority women and broader LGBTQ populations. In these studies, sexual orientation was examined alongside weight stigma either descriptively or through subgroup or interaction-based analyses. Socioeconomic position or class was examined in 7 studies, most commonly operationalized using income, education, subjective socioeconomic status, or class-based indicators.

A smaller number of studies examined additional axes, including disability status or chronic illness (*n* = 4), age or age-related marginalization (*n* = 3), immigration status (*n* = 2), and skin color (*n* = 1). Across studies, most examined two or more intersectional axes in relation to weight stigma, with the number and combination of axes varying by study.

### Methodological Implementation of Intersectionality

Studies varied in the analytic approaches used to examine intersecting social identities in relation to weight stigma (Table 2).

Across the ten qualitative studies, intersectionality was operationalized through interpretive analyses of participants’ narratives, including reflexive thematic analysis, phenomenological analysis, life history–informed approaches, and one autoethnographic study. These analyses examined how experiences of weight stigma were articulated in relation to multiple social identities, as described by study authors. For example, a study applied a life history–informed, reflexive thematic analytic approach in which intersectionality guided analytic decisions, examining weight stigma as it unfolded over the life course of sexual minority women, with interpretive attention to how sexual orientation and body size intersected with participants’ racialized and gendered experiences [32].

Six studies used interaction-based regression models, testing interaction terms between weight-related variables and other social identities, such as race or sexual orientation. For instance, one study operationalized intersectionality by estimating interaction terms between weight-based discrimination and race/ethnicity and household income, testing whether the mental health consequences of discrimination were conditioned by respondents’ racial/ethnic and socioeconomic positions [23].

Three studies used stratified or subgroup-based comparisons to assess differences in weight stigma across predefined social identity groupings. For example, one study compared experienced and internalized weight stigma between sexual minority and heterosexual women matched on age, race, and BMI in a U.S. registry sample, operationalizing intersectionality through comparative group design, positioning sexual orientation as the intersecting axis with weight stigma [17].

One study operationalized intersectionality among ethnically diverse U.S. adolescents by applying latent class analysis to identify person-centered patterns of co-occurring attributed discrimination and bullying, based on race/ethnicity, immigration status, perceived sexual orientation, and weight within a school-based survey sample [27].

Another quantitative study longitudinally examined intersectionality by modeling accounts of discrimination experiences attributed to race/ethnicity, perceived sexual orientation, and weight among U.S. children aged 9–12 years, and estimating these associations separately for youth with no, single and intersecting marginalized identities, including overweight status [18].

### Outcomes and Phenomena Examined

Across study designs, outcomes and phenomena reflected a range of individual-, interpersonal-, and institutional-level experiences related to weight stigma.

Among qualitative studies, the primary phenomena examined were lived experiences of weight stigma and its co-occurrence with other forms of stigma. These studies focused on stigma in healthcare settings, family and social contexts, public spaces, and across the life course. They also examined how such experiences shaped healthcare access and interactions, identity development, body image, social belonging, and everyday interactions, including the navigation of sexual relationships [20, 28, 30].

Among the quantitative studies, the most commonly examined outcomes were mental health–related outcomes, including psychological distress, depressive symptoms, perceived stress, and eating-related psychopathology [1, 23, 24, 33]. Several studies also examined internalized weight stigma or experienced weight-based discrimination as primary outcomes [1, 17, 25]. Additional quantitative outcomes included substance use intention [18], self-rated health [33], and coping responses to weight stigma [1]. One study examined public obesity stigma outcomes, including fat phobia, emotional reactions, and desire for social distance toward hypothetical targets [22].

### Key Intersectional Findings Across Studies

Across the included studies, intersectional findings are clustered into four themes: (1) co-occurring forms of stigma across identities; (2) institutional and context-specific intersectional findings; (3) group differences in weight stigma; and (4) cumulative intersectional disadvantage. The findings are summarized below with emphasis on patterns observed across studies.

#### Co-occurring Forms of Stigma Across Identities

Nine studies reported intersectional findings describing weight stigma as occurring alongside other forms of stigma or marginalization, based primarily on qualitative accounts [20, 29, 31, 32, 36]. In these studies, participants described experiences of weight stigma as intertwined with other stigmatized identities, including race and ethnicity, gender identity, sexual orientation, and body size.

Reported findings included descriptions of weight stigma occurring simultaneously with racism, cissexism, ableism, classism, or heteronormativity in everyday interactions and institutional encounters [21, 28, 32]. Several studies described how fatness, in combination with other marginalized identities shaped expectations around body norms, attractiveness, or social belonging, including within queer communities and racialized contexts [29, 30, 34]. Other studies described weight stigma as embedded within broader systems of oppression, with experiences framed through multiple intersecting stigmatized identities rather than as isolated forms of discrimination [20, 36].

Across studies, these co-occurring stigmas shaped experiences of healthcare access and quality. Participants described delayed or avoided care due to anticipated discrimination as well as clinical encounters in which symptoms were attributed to body weight rather than investigation, particularly among individuals with multiple marginalized identities [20, 28].

Beyond healthcare, intersecting stigmas shaped social and relational experiences, including perceptions of attractiveness, desirability, and belonging. Participants reported appearance-based scrutiny, exclusion, and conflicting body norms across family, community, and sexual contexts, reflecting the combined influence of multiple stigmatized identities on everyday interactions [29, 30, 32].

In summary, weight stigma was described as operating alongside other forms of marginalization, shaping healthcare experiences, social belonging, and everyday interactions rather than appearing as an isolated form of discrimination.

#### Institutional and Context-Specific Intersectional Findings

Several qualitative studies reported institution-specific manifestations of intersectional weight stigma, most frequently within healthcare settings, where participants described how clinical interactions, treatment decisions, and access to care varied across intersecting social identities [20, 21, 36]. In healthcare contexts, participants reported that symptoms and pain were frequently attributed to body weight, particularly among Black participants, gender-diverse individuals, and those with additional marginalized identities. These accounts included experiences of delayed evaluation, dismissal of concerns, or denial of care, including gender-affirming or sexual and reproductive health services [20, 28, 36].

Beyond healthcare contexts, qualitative studies described intersectional weight stigma within family environments, public spaces, educational settings, and queer or LGBTQ+ communities, where participants reported appearance-based scrutiny, exclusion, or conflicting body norms linked to body size alongside other social identities [19, 21, 29, 32].

In addition, one qualitative study examined intersectional weight stigma within casual sexual encounters among larger-bodied sexual minority men, finding that intra-minority norms and fetishization produced anticipatory stigma that constrained partner selection, shaped stigma management strategies, and altered experiences of sexual safety and embodied pleasure during sexual interactions [30].

These studies suggest that intersectional weight stigma manifested differently across institutional and social contexts, with healthcare settings emerging as key sites where intersecting identities shaped access to care, clinical decision-making, and patient experiences.

#### Group Differences in Weight Stigma

Nine quantitative studies examined differences in weight stigma and related health outcomes across intersecting social identities, including race, gender, sexual orientation, and socioeconomic position [1, 17, 22–26, 35]. Intersectional differences were most often observed in terms of internalized weight stigma and associations between weight-based discrimination and mental health outcomes. Race- and gender-related variation was reported most frequently, with some studies finding lower internalized weight stigma among Black participants than White participants, particularly among women, while others reported higher levels among Black participants or no differences, depending on the identities and outcomes examined [1, 25, 26].

Sexual minority individuals generally reported higher levels of both experienced and internalized weight stigma than heterosexual individuals [17, 25].

Lower socioeconomic position was also associated with stronger adverse mental health associations with weight-based discrimination, whereas higher socioeconomic position attenuated these associations [22, 23]. However, interaction effects were not consistently observed across all studies, with some analyses reporting no significant differences by gender, race/ethnicity, age, or socioeconomic status [22, 33, 35].

Overall, intersectional differences in weight stigma were context-dependent and did not follow uniform patterns across groups.

#### Cumulative Intersectional Disadvantage

One quantitative study reported evidence of cumulative intersectional disadvantage [18]. In this longitudinal study of U.S. youth, participants were categorized into non-marginalized, single-marginalized, and intersecting-marginalized identity groups based on race/ethnicity, sexual orientation, and overweight status. Youth with intersecting marginalized identities reported higher levels of multiple discrimination and greater substance-use intention than those with no or single marginalized identities. Longitudinal analyses further indicated that multiple discrimination predicted subsequent substance-use intention only among youth with intersecting marginalized identities, with greater exposure associated with higher substance-use intention over time.

In all, the key findings of the included studies indicate that weight stigma is embedded within co-occurring systems of marginalization rather than operating as an isolated experience. Across studies, these intersecting stigma processes shaped both everyday social experiences and access to care, while their consequences varied across social identities, indicating that the effects of weight stigma are not uniform across populations. Healthcare settings emerged as a particularly important institutional context in which these intersecting forms of stigma were expressed, influencing clinical interactions, patient experiences, and access to care. These findings are consistent with recent evidence demonstrating that weight stigma in healthcare settings contributes to delayed care, avoidance of healthcare services, and poorer patient–provider experiences [7, 8].

## Discussion

### Empirical Application of Intersectionality

This systematic review examined how intersectionality is conceptualized and operationalized in empirical studies of weight stigma, including its theoretical grounding, analytic strategies and measurement choices. Through synthesis of qualitative and quantitative evidence, this review provides a focused methodological reflection of empirical application of intersectionality in the context of weight stigma, highlighting patterns of theoretical alignment, analytic implementation, and variation across studies.

One of the central insights emerging from this review concerns the distinction between explicitly referencing intersectionality and embedding it within analytic practice. Across studies, intersectionality was commonly articulated as part of study framing, often informing research aims, sampling strategies, and interpretive narratives [1, 17, 32]. This reflects an important shift away from single-axis approaches and highlights recognition that weight stigma is experienced in relation to other forms of marginalization rather than in isolation. However, the extent to which intersectionality explicitly shaped analytic decisions varied considerably, particularly in quantitative research [26, 27, 33]. Overall, intersectionality was more consistently used as a conceptual framing tool than as an analytic framework, reflecting both long-standing and ongoing theoretical and methodological debates about how to operationalize it within empirical health research [9, 37, 38].

Methodological approach played a central role in how intersectionality was operationalized within empirical analyses. In qualitative studies, intersectionality was commonly integrated across multiple stages of the research process, including data collection, analytic interpretation, and presentation of findings, allowing for examination of how weight stigma was experienced in relation to intersecting social positions and institutional contexts [30, 36]. In contrast, quantitative studies most often operationalized intersectionality through interaction terms, stratified analyses, or cumulative measures of discrimination [17, 18, 23]. These strategies were generally used to either assess whether associations involving weight stigma differed across social groups or to examine whether exposure to multiple forms of discrimination was associated with health-related outcomes.

While these approaches provide important insights, they also reveal key limitations in how intersectionality has been operationalized. In quantitative studies, intersectionality was frequently reduced to identity-based comparisons or effect modification, which emphasized differences between social groups more than the joint operation of multiple stigma processes. As a result, these approaches were less able to capture how intersecting systems of power shape experiences of weight stigma. In qualitative studies, although intersectionality was well suited to examining meaning and context and was often integrated across research stages, many analyses remained primarily descriptive, focusing on co-occurring forms of stigma without clearly tracing how those intersections produced distinct experiences or outcomes. In addition, qualitative studies tended to emphasize interpersonal experiences rather than structural or institutional processes and were often conducted with small, demographically narrow samples.

Quality appraisal indicated that the majority of included studies met established methodological standards, with most quantitative studies rated as fair to good quality using the NIH assessment tool and all qualitative studies demonstrating strong alignment between research aims, methodology, and analytic approach based on CASP criteria. Common limitations among quantitative studies included limited reporting of sample size justification and reliance on cross-sectional designs. These limitations reflect prevailing conventions in the field, although they remain relevant when interpreting the strength of the evidence. Importantly, these quality assessments evaluated general methodological rigor but did not assess the extent to which intersectionality was theoretically integrated or analytically operationalized. As a result, variation in how intersectionality was conceptualized and operationalized was observed even among studies rated as methodologically strong. This highlights the limitations of conventional quality appraisal tools for evaluating intersectional rigor and reinforces the need for theory-informed approaches to assessing intersectionality in empirical research.

### Knowledge Gaps

Several gaps and imbalances in existing literature warrant attention. Nearly all included studies were conducted in Western contexts, with the majority based in the United States, limiting insight into how intersectional weight stigma operates across different sociopolitical contexts and systems. Certain axes of marginalization, including immigration status, skin color, disability, and structural class position, were examined infrequently relative to race, gender, and sexual orientation. In addition, longitudinal and system-level analyses were uncommon, constraining understanding of how intersectional weight stigma unfolds over time and across organizational and policy contexts. Relatedly, no studies explicitly examined how institutional policies, healthcare systems, and broader sociopolitical structures shaped the production and maintenance of intersectional weight stigma. These gaps limit the field’s ability to examine how intersecting stigma processes accumulate, interact, and change over time and across institutional and structural contexts [13, 37].

### Recommendations for Future Research

These gaps point to several priorities for advancing intersectional weight stigma research. First, studies should move beyond identity-based comparisons to examine co-occurring stigma processes, including how weight stigma operates alongside racism, sexism, and other forms of marginalization. Second, greater alignment between theoretical frameworks, measurement, and analytic strategies is needed to ensure that intersectionality is operationalized rather than only conceptually referenced. This includes developing measures and analytic approaches that capture the relational and mutually constitutive nature of intersecting systems of power. Where appropriate, quantitative studies may also benefit from approaches such as multilevel modeling and Multilevel Analysis of Individual Heterogeneity and Discriminatory Accuracy (MAIHDA), which can support more explicit examination of intersecting social positions without reducing intersectionality to single-axis group comparisons [39, 40]. Third, longitudinal and multilevel study designs are needed to examine how intersectional weight stigma unfolds over time and across institutional and policy contexts. Finally, integrating qualitative and quantitative approaches through mixed-methods designs may strengthen the field by combining detailed accounts of lived experience with the ability to assess variation and impact across populations.

### Implications for Intervention and Policy

These findings indicate that weight stigma should not be addressed as a single-axis issue, because its forms and consequences vary across intersecting social identities. Applying an intersectional lens in public health practice is necessary to ensure that interventions and policies reflect the lived experiences of populations most affected by stigma. Healthcare settings represent a critical site where weight stigma is enacted, highlighting the need for greater attention to provider–patient communication and the ethical use of weight-related language in clinical care. Without such approaches, efforts to reduce weight stigma may fail to address its contribution to inequities in access to care and health outcomes.

### Strengths and Limitations of the Current Review

This review has several strengths, including its explicit focus on intersectionality as an analytic construct, inclusion of both qualitative and quantitative evidence, and attention to how intersectionality is translated into empirical practice. Several limitations should also be acknowledged. Restricting inclusion to English-language, peer-reviewed studies and to studies that explicitly referenced intersectionality may have excluded relevant work that engaged intersectional concepts implicitly or used alternative terminology.

## Conclusion

This review highlights a fundamental tension in intersectional weight stigma research between theoretical recognition and empirical implementation. While intersectionality is widely acknowledged as essential for understanding weight stigma, its translation into analytic practice remains inconsistent across studies. This disconnect shapes how weight stigma is conceptualized, measured, and interpreted, highlighting the need for approaches that more clearly align analytic strategies with intersectionality’s theoretical foundations. Addressing this tension will be critical for advancing intersectionality as a meaningful analytic framework in weight stigma research.

## Key References

- Bowleg L. The problem with the phrase women and minorities: intersectionality—an important theoretical framework for public health. *Am J Public Health*.2012.102(7):1267-73
  - ◦ This article critiques public health research that treats social categories such as race or gender independently and argues that such approaches obscure the complex intersections of identity and structural inequality that shape health disparities. It proposes intersectionality as a theoretical framework for examining how multiple social identities interact to influence health outcomes.
- Bauer GR et al. Intersectionality in quantitative research: A systematic review of its emergence and applications of theory and methods. *SSM Popul Health*. 2021.14:100798
  - ◦ This systematic review examines how intersectionality has been applied in quantitative health research and highlights persistent challenges in translating intersectional theory into empirical analytic strategies.
- Harari, L. and C. Lee, Intersectionality in quantitative health disparities research: A systematic review of challenges and limitations in empirical studies. *Soc Sci Med*, 2021. 277:113876.
  - ◦ This review examines limitations in the application of intersectionality in quantitative health disparities research and offers recommendations to improve the integration of intersectional theory with empirical analyses.
- Pearl RL and Sheynblyum M. How weight bias and stigma undermine healthcare access and utilization. *Current Obesity Reports.* 2025;14(1):11.
  - ◦ This review synthesizes evidence indicating that weight stigma in healthcare settings contributes to healthcare avoidance, delayed care seeking, and poorer patient experiences among individuals with higher body weight.
- Ryan L, et al. Weight stigma experienced by patients with obesity in healthcare settings: A qualitative evidence synthesis. *Obesity Reviews.* 2023;24(10):e13606.
  - ◦ This qualitative evidence synthesis examines how patients experience weight stigma in healthcare settings and shows how stigmatizing interactions with healthcare providers influence the provision of care, shape patient–provider relationships, and create barriers to equitable healthcare access.
- Puhl, R.M., M.S. Himmelstein, and R.L. Pearl, Weight stigma as a psychosocial contributor to obesity. *Am Psychol*, 2020.75(2):274-289.
  - ◦ This article reviews evidence linking experiences of weight stigma to weight-related behaviors and health outcomes.
- Lapalme, J., R. Haines-Saah, and K.L. Frohlich, More than a buzzword: how intersectionality can advance social inequalities in health research. Critical Public Health, 2020. 30(4): p. 494-500.
  - ◦ This article discusses how intersectionality can advance research on social inequalities in health and argues for empirical approaches that examine both structural forces and the lived experiences of marginalized groups.
- Himmelstein MS et al. Intersectionality: An understudied framework for addressing weight stigma. *Am J Prev Med*. 2017.53(4):421-431
  - ◦ This study demonstrates the importance of intersectional analyses in weight stigma research by examining how race and gender jointly shape stigma internalization and coping responses.
- Agenor, M., Tipnis, A., Byers, M., Davis, T., Noh, M., Dunham, R., Reyna, K., Adesuyi, Q., Bazzi, A. R., & Biello, K. B. (2025). Structural barriers to sexual and reproductive health care among Black and Latina cisgender and transgender U.S. women who use drugs: a qualitative study. *BMC Health Serv Res*,* 25*(1), 754.
  - ◦ This study provides a structurally grounded application of intersectionality, demonstrating how multiple systems of oppression shape healthcare access and quality, resulting in delayed or avoided care among Black and Latina women who use drugs.
- Fowler, L. A., Wang, Y. L., Wall, C., Velkovich, A., Harrop, E. N., Vázquez, M. M., Mensah, J., Flentje, A., & Mann, E. S. (2025). "If I can accept my queerness, I can accept my body as it is": Understanding weight-related perspectives and stigma from sexual minority women. *Frontiers in psychiatry*,* 16*, 1687680.
  - ◦ This study conceptualizes weight stigma as a cumulative and contextually embedded process, produced through interlocking systems of oppression, that operate across cultural, familial, and queer community contexts to shape body image, mental health, and disordered eating over the life course.

## Supplementary Information

Below is the link to the electronic supplementary material.

Supplementary Material 1

## Data Availability

All data analyzed in this review were derived from studies included in the published literature. Extracted data are presented in the manuscript and supplementary materials.

## Abbreviations

CASP: Critical Appraisal Skills Programme
MeSH: Medical Subject Headings
NIH: National Institutes of Health
PRISMA: Preferred Reporting Items for Systematic Reviews and Meta-Analyses
PROSPERO: International Prospective Register of Systematic Reviews
