# Supplementary material for: Intersectionality in Weight Stigma Research: A Systematic Review of Empirical Evidence
Source: Curr Obes Rep. 2026 Apr 15;15(1):35. doi: 10.1007/s13679-026-00715-6 (PMC13079528; doi:10.1007/s13679-026-00715-6)
Supplement: Supplementary file 1 — Supplementary Material 1 [file 13679_2026_715_MOESM1_ESM.docx]

**Supplementary File 1. Full PubMed electronic search strategy (search conducted December 12, 2025)**

(("Weight Prejudice"[Mesh] OR "weight stigma*"[tiab] OR "weight-related stigma*"[tiab] OR "weight prejudice*"[tiab] OR "fat bias*"[tiab] OR "obesity bias*"[tiab] OR "obesity stigma*"[tiab] OR "fat phobi*"[tiab] OR fatphobi*[tiab] OR "weight bias*"[tiab] OR "anti-fat bias*"[tiab] OR "fat shaming"[tiab] OR "weight-based discriminat*"[tiab] OR "weight-based stigma*"[tiab] OR "weight discriminat*"[tiab] OR "weight victim*"[tiab] OR "weight bulling"[tiab] OR "weight teasing"[tiab] OR "weight prejudice*"[tiab] OR sizeism[tiab] OR weightism[tiab]) AND ((("Intersectional Framework"[Mesh] OR intersection*[tiab] OR intersecting[tiab] OR "multiple layer*"[tiab] OR overlap*[tiab] OR "interaction* between"[tiab] OR "combined effect*"[tiab] OR "moderated by"[tiab] OR "joint effect*"[tiab] OR "cumulative discrimination"[tiab] OR "multiple stigma*"[tiab] OR "double discrimination"[tiab] OR "compound* discrimination"[tiab]) OR ("Sexual and Gender Minorities"[Mesh] OR "Bisexuality"[Mesh] OR "Homosexuality"[Mesh] OR "Transsexualism"[Mesh] OR homosexual*[tiab] OR "sexual minorit*"[tiab] OR "gender minorit*"[tiab] OR transgender*[tiab] OR transsexual*[tiab] OR bisexual*[tiab] OR bigender*[tiab] OR gay[tiab] OR gays[tiab] OR "gender divers*"[tiab] OR LGBT*[tiab] OR GLBT*[tiab] OR lesbian*[tiab] OR "men who have sex with men"[tiab] OR "same sex"[tiab] OR homophobia OR transphobia[tiab])) OR ("Minority Groups"[Mesh] OR "Racial Groups"[Mesh:NoExp] OR "American Indian or Alaska Native"[Mesh] OR "African People"[Mesh] OR "Asian People"[Mesh] OR "Black People"[Mesh] OR "Native Hawaiian or Pacific Islander"[Mesh] OR minorit*[tiab] OR racism[tiab] OR "men of color"[tiab] OR "men of colour"[tiab] OR "women of color"[tiab] OR "women of colour"[tiab] OR "patient* of color"[tiab] OR "patient* of colour"[tiab] OR "person* of color"[tiab] OR "person* of colour"[tiab] OR "people of color"[tiab] OR "people of colour"[tiab] OR BIPOC[tiab] OR ethnic*[tiab] OR racial[tiab] OR "mixed race"[tiab] OR biracial[tiab] OR multi-ethnic*[tiab] OR black*[tiab] OR african*[tiab] OR hispanic*[tiab] OR hispano*[tiab] OR hispana*[tiab] OR latino*[tiab] OR latinx[tiab] OR latine*[tiab] OR indigenous[tiab] OR native*[tiab] OR tribe*[tiab] OR tribal[tiab] OR "pacific islander*"[tiab] OR asian*[tiab]))) AND ("Health Inequities"[Mesh:NoExp] OR "Health Status Disparities"[Mesh] OR "Healthcare Disparities"[Mesh:NoExp] OR "Health Equity"[Mesh] OR "Health Services Accessibility"[Mesh:NoExp] OR "Health Equity"[Mesh] OR "Weight Loss"[Mesh:NoExp] OR "Weight Cycling"[Mesh] OR "Body Weight Maintenance"[Mesh] OR "mental health"[tiab] OR "mental wellbeing"[tiab] OR "mental well-being"[tiab] OR "mental distress"[tiab] OR "emotional distress"[tiab] OR psychological[tiab] OR stress*[tiab] OR depress*[tiab] OR anxiet*[tiab] OR disparit*[tiab] OR inequit*[tiab] OR inequalit*[tiab] OR equit*[tiab] OR "healthcare quality"[tiab] OR "care quality"[tiab] OR "healthcare access*"[tiab] OR "care access*"[tiab] OR "access* to care"[tiab] OR "health behavior*"[tiab] OR "health behaviour*"[tiab] OR "weigh loss"[tiab] OR "weight reduction*"[tiab] OR "weight maintenance"[tiab] OR "weight control"[tiab] OR "weight change*"[tiab] OR "weight cycling"[tiab]) AND 2000:2025[dp] AND eng[la]

**Supplementary Table S1. National Institutes of Health (NIH) Quality Assessment Tool for Observational Cohort and Cross-Sectional Studies assessment of included quantitative studies**

| **Criteria** | Adams, 2025 | Beccia et al., 2020 | Ciciurkaite & Perry, 2018 | Garnett et al., 2014 | Gerend et al., 2024 | Himmelstein et al. 2017 | Launius & Lydecker, 2024 | Makowski et al., 2019 | Osa et al., 2025 | Panza et al., 2024 | Wang et al., 2024 |
| --- | --- | --- | --- | --- | --- | --- | --- | --- | --- | --- | --- |
| 1. Was the research question or objective in this paper clearly stated? | Yes | Yes | Yes | Yes | Yes | Yes | Yes | Yes | Yes | Yes | Yes |
| 2. Was the study population clearly specified and defined? | Yes | Yes | Yes | Yes | Yes | Yes | Yes | Yes | Yes | Yes | Yes |
| 3. Was the participation rate of eligible persons at least 50%? | NR | Yes | Yes | Yes | NR | NR | NR | No | NR | NR | NR |
| 4. Were all the subjects selected or recruited from the same or similar populations (including the same time period)? Were inclusion and exclusion criteria for being in the study prespecified and applied uniformly to all participants? | Yes | Yes | Yes | Yes | Yes | Yes | Yes | Yes | Yes | Yes | Yes |
| 5. Was a sample size justification, power description, or variance and effect estimates provided? | No | No | No | No | No | No | No | No | No | No | No |
| 6. For the analyses in this paper, were the exposure(s) of interest measured prior to the outcome(s) being measured? | No | No | No | No | No | No | No | No | No | No | Yes |
| 7. Was the timeframe sufficient so that one could reasonably expect to see an association between exposure and outcome if it existed? | No | No | No | No | No | No | No | No | No | No | Yes |
| 8. For exposures that can vary in amount or level, did the study examine different levels of the exposure as related to the outcome (e.g., categories of exposure, or exposure measured as continuous variable)? | Yes | Yes | Yes | Yes | Yes | Yes | Yes | Yes | Yes | Yes | Yes |
| 9. Were the exposure measures (independent variables) clearly defined, valid, reliable, and implemented consistently across all study participants? | Yes | Yes | Yes | Yes | Yes | Yes | Yes | Yes | Yes | Yes | Yes |
| 10. Was the exposure(s) assessed more than once over time? | NA | NA | NA | NA | NA | NA | NA | NA | NA | NA | Yes |
| 11. Were the outcome measures (dependent variables) clearly defined, valid, reliable, and implemented consistently across all study participants? | Yes | Yes | Yes | Yes | Yes | Yes | Yes | Yes | Yes | Yes | Yes |
| 12. Were the outcome assessors blinded to the exposure status of participants? | No | No | No | No | No | No | No | No | No | No | No |
| 13. Was loss to follow-up after baseline 20% or less? | NA | NA | NA | NA | NA | NA | NA | NA | NA | NA | Yes |
| 14. Were key potential confounding variables measured and adjusted statistically for their impact on the relationship between exposure(s) and outcome(s)? | Yes | Yes | Yes | Yes | Yes | Yes | No | Yes | Yes | Yes | Yes |
| **Rating** | Fair | Good | Good | Good | Fair | Fair | Poor | Fair | Fair | Fair | Good |

*Each criterion was rated as Yes, No, Not Reported (NR), or Not Applicable (NA).*

**Supplementary Table S2. Critical Appraisal Skills Programme (CASP) checklist assessment of included qualitative studies**

| **Criteria** | Agénor et al., 2022 | Agénor et al., 2025 | Airhart-Larraga et al., 2025 | Biefeld et al., 2025 | Deol et al., 2024 | Elbe et al., 2024 | Fowler et al., 2025 | Gerend et al., 2022 | Harrop & Kattari, 2022 | Paine, 2021 |
| --- | --- | --- | --- | --- | --- | --- | --- | --- | --- | --- |
| 1. Was there a clear statement of the aims of the research? | Yes | Yes | Yes | Yes | Yes | Yes | Yes | Yes | Yes | Yes |
| 2. Is a qualitative methodology appropriate? | Yes | Yes | Yes | Yes | Yes | Yes | Yes | Yes | Yes | Yes |
| 3. Was the research design appropriate to address the aims of the research? | Yes | Yes | Yes | Yes | Yes | Yes | Yes | Yes | Yes | Yes |
| 4. Was the recruitment strategy appropriate to the aims of the research? | Yes | Yes | Yes | Yes | Yes | Yes | Yes | Yes | Not Applicable | Yes |
| 5. Was the data collected in a way that addressed the research issue? | Yes | Yes | Yes | Yes | Yes | Yes | Yes | Yes | Yes | Yes |
| 6. Has the relationship between researcher and participants been adequately considered? | Yes | No | Yes | Yes | Yes | Yes | Yes | Yes | Yes | Yes |
| 7. Have ethical issues been taken into consideration? | Yes | Yes | Yes | Yes | Yes | Yes | Yes | Yes | Can't tell | Yes |
| 8. Was the data analysis sufficiently rigorous? | Yes | Yes | Yes | Yes | Yes | Yes | Yes | Yes | Can't tell | Yes |
| 9. Is there a clear statement of findings? | Yes | Yes | Yes | Yes | Yes | Yes | Yes | Yes | Yes | Yes |
| 10. How valuable is the research? | Yes | Yes | Yes | Yes | Yes | Yes | Yes | Yes | Yes | Yes |

*Assessments were conducted using the CASP qualitative checklist. Responses were coded as Yes, No, Can’t tell, or Not applicable based strictly on information explicitly reported in each article*
